# Comparison of the effectiveness of extracorporeal shock wave therapy and high-intensity laser therapy in patients with knee osteoarthritis: a single-blind randomized clinical trial

**DOI:** 10.1007/s10067-026-08052-8

**Published:** 2026-03-29

**Authors:** Orge Fatos Demirtas, Ozlem Altindag, Mazlum Serdar Akaltun, Neytullah Turan, Elif Balbal, Ali Gur

**Affiliations:** Department of Physical Medicine and Rehabilitaton, Faculty of Medicine, Gaziantep University, Gaziantep, Turkey

**Keywords:** Extracorporeal shock wave therapy, Laser therapy, Osteoarthritis of knee

## Abstract

**Objective:**

The aim of this single-blind randomized clinical trial was to compare the effectiveness of extracorporeal shock wave therapy (ESWT) and high-intensity laser therapy (HILT) on clinical parameters in knee osteoarthritis.

**Patients and methods:**

A total of 60 patients aged between 40 and 75 years, diagnosed with primary knee osteoarthritis and admitted to the Department of Physical Medicine and Rehabilitation, Gaziantep University, were included in the study. Sixty patients were randomized into two groups using the envelope method. Group 1 received ESWT (3 sessions/week, total of 6 sessions), and Group 2 received HILT (5 sessions/week, total of 10 sessions) for a duration of 2 weeks. A standardized home exercise program was applied to all patients. Patients were evaluated before treatment, after treatment, and at the 6th week post-treatment using the Visual Analog Scale (VAS), Western Ontario and McMaster Universities Osteoarthritis Index (WOMAC), and the Lequesne algofunctional knee index. Statistical analyses were performed using SPSS 22.0 (IBM Corp., Armonk, NY, USA), including *t*-test, chi-square, ANOVA, and Pearson correlation; significance was set at *p* < 0.05.

**Results:**

In both groups, compared to pre-treatment values, significant improvements were observed in VAS (*p* < 0.001), WOMAC pain, stiffness, and physical function (*p* < 0.001), as well as Lequesne index scores (*p* < 0.001) after treatment and at the 6th week post-treatment. No significant differences were found between the groups in intergroup comparisons.

**Conclusion:**

Both ESWT and HILT are effective and safe treatment methods for reducing pain, disease severity, and improving physical function in knee osteoarthritis. Our findings support broader clinical use of both treatments, though further comprehensive studies are required.

**Table:** 

**Key Points** *• In both the ESWT and laser groups, significant improvements were observed in VAS, WOMAC pain, stiffness, and physical function and Lequesne index scores at 6 weeks post-treatment compared to pre-treatment values.* *• In patients with knee osteoarthritis receiving ESWT and HILT treatment, improvement was achieved in terms of pain and functionality, but our study showed that neither agent was superior to the other.*

## Introduction

Osteoarthritis (OA) is a degenerative joint disease characterized by erosion of the articular cartilage, bone hypertrophy (osteophytes) at the joint margins, subchondral sclerosis, and biochemical and morphological changes in the synovial membrane and joint capsule and involves all joint tissues, including the meniscus and the infrapatellar fat pad [1, 2]. The etiology of osteoarthritis involves both general risk factors—such as advanced age, female sex, genetic predisposition, and endocrine disorders—and local risk factors, including obesity, trauma (e.g., tibial plateau fractures), joint structural characteristics, occupational factors, certain physical activities, and quadriceps weakness [3]. OA results in pain, joint stiffness, immobility, limitations in daily living activities, and ultimately disability [4]. Although osteoarthritis can affect all joints, it most commonly affects the knee, hip, hand, and spine joints [5].

The goal of osteoarthritis treatment is to reduce pain, stop further damage to the joint, and educate the patient about osteoarthritis to improve their physical functioning and quality of life. Although there is no curative treatment for OA, many methods are recommended for symptomatic treatment. Pharmacological treatment methods include nonsteroidal anti-inflammatory drugs (NSAIDs), and analgesic drugs (acetaminophen and opioids) are recommended in many treatment guidelines. Exercise contributes to functional improvement by increasing muscle strength and flexibility [6, 7]. Many non-pharmacological treatment methods, such as magnetic field therapy, transcutaneous electrical nerve stimulation, and therapeutic ultrasound, are frequently used today. Previous studies have shown that adding physical therapy agents to exercise therapy in OA improves clinical outcomes [8].

Extracorporeal shock wave therapy (ESWT) is a non-invasive treatment modality that delivers high-energy acoustic waves to musculoskeletal tissues, triggering a cascade of biological responses rather than producing purely mechanical effects. Experimental and clinical studies suggest that ESWT exerts its therapeutic action primarily through mechanotransduction, whereby mechanical stimuli are converted into cellular and molecular signals [9]. ESWT affects inflammatory processes and accelerates bone repair. It also activates numerous cellular processes necessary for tissue regeneration and neovascularization [10]. In their studies, Wang et al. demonstrated that shock waves stimulate the early expression of growth factors related to angiogenesis, thereby promoting cell renewal and bone and tendon repair [11]. In recent years, studies have shown that ESWT has a positive effect on pain and clinical findings in OA [12].

High-intensity laser therapy (HILT) is a treatment method that has become increasingly popular in recent years. It is non-invasive and more powerful than low-intensity laser therapy, capable of affecting deeper tissues. HILT increases local blood circulation, improves tissue regeneration, and reduces pain and edema [13]. HILT can be used in many painful conditions due to its biostimulation, analgesic, and anti-inflammatory effects [14, 15]. The aim of this study is to compare the effectiveness of ESWT and HILT treatments on clinical findings in patients with knee osteoarthritis.

Although both ESWT and HILT have been shown to be effective in reducing pain and improving function in patients with knee osteoarthritis, the existing literature largely evaluates these modalities independently. Direct comparative studies between ESWT and HILT are limited, and differences in treatment protocols, outcome measures, and follow-up durations make it difficult to draw firm conclusions regarding their relative effectiveness. Moreover, the two modalities act through distinct biological mechanisms, suggesting that they may lead to different clinical outcomes. This highlights an important gap in the current literature and underscores the need for direct comparative research.

## Patients and methods

This study was designed as a single-blind randomized clinical trial. Prior to the study, the Gaziantep University Clinical Research Ethics Committee approved the study protocol (approval no: 2020/27). The study was conducted in accordance with the principles of the Declaration of Helsinki. Written and verbal informed consent was obtained from all patients included in the study prior to their participation.

### Participants

Patients aged 40–75 years with a diagnosis of knee OA were included in the study. The American College of Rheumatology (ACR) classification criteria were used for the diagnosis of OA. Inclusion criteria for the study were as follows: (1) diagnosis of primary knee osteoarthritis according to the American College of Rheumatology criteria; (2) stage 2–3 knee OA cases according to the Kelgren-Lawrence classification; (3) being between 40 and 75 years of age; (3) providing consent to participate in the study; (4) having knee pain for at least 6 months; (5) having a VAS score of at least 3 or higher. 

Exclusion criteria were as follows: (1) those with inflammatory arthritis (such as rheumatoid arthritis, spondyloarthropathies); (2) history of knee surgery or trauma; (3) intra-articular knee injections within the last 6 months; (4) history of cancer, bleeding diathesis, systemic inflammatory disease; (5) pregnancy; (6) participants who had participated in another physical therapy program within the last 6 months were excluded from the study.

### Randomization

**Fig. 1:**
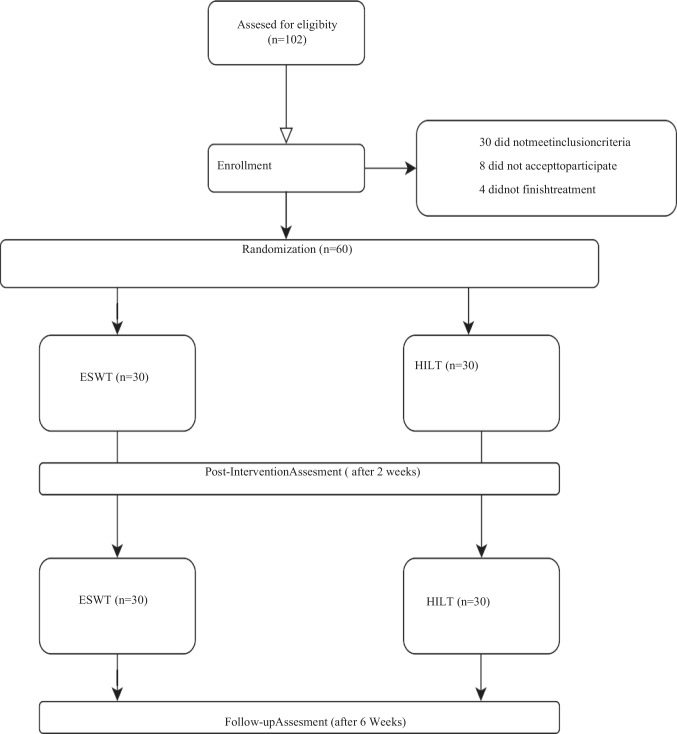
Flowchart of study

Initially, 102 patients were included in this study. Thirty patients did not meet the inclusion criteria, eight patients refused to participate in the treatment, and four patients were unable to complete the treatment. Sixty patients were randomized into two groups using the envelope method. Group 1 received HILT therapy (*n* = 30), and Group 2 received ESWT therapy (*n* = 30) (Fig. 1).

### Assessment tools

Sociodemographic data such as age, gender, and body mass index were recorded for all patients included in the study. After taking a detailed history and performing a musculoskeletal examination, routine blood tests, a complete blood count, CRP, erythrocyte sedimentation rate, and knee radiographs were requested from each patient. Radiological evaluation was performed based on standing anterior–posterior and lateral knee radiographs and the Kellgren-Lawrence classification [16]. Evaluations were performed by the same investigator, who was unaware of the treatment groups, before treatment, immediately after treatment (at 2 weeks), and after treatment (at 6 weeks).

### Pain assessment

Pain was assessed using the Visual Analog Scale (VAS). Absence of pain was scored as 0 points, and the most severe pain was scored as 10 points [17].

### Functional assessment

The functional status of patients was assessed using the WOMAC (Western Ontario and McMaster Universities Arthritis Index) and the Lequesne index. WOMAC is a self-administered disease-specific measure for patients with knee and hip OA and consists of a total of 24 items in three groups: pain, function, and stiffness. Each question is scored from 0 to 4. The Turkish version of the WOMAC questionnaire has been shown to be valid and reliable, with acceptable internal consistency (Cronbach’s alpha = 0.70), as reported by Tüzün et al. [18].

The Lequesne index is another index that evaluates pain, stiffness, and functionality in patients with knee osteoarthritis. Pain or discomfort, maximum walking distance, and daily living activities are grouped under three separate headings. The Lequesne index results in a value between 0 and 24 points. The validity and reliability of the Turkish version of the Lequesne knee index were demonstrated by Basaran et al., with a reported Cronbach’s alpha coefficient of 0.70 [19].

### Treatment protocol

ESWT (EMS-SwissDolorclast Master) was administered 3 sessions per week for 2 weeks. ESWT treatment was applied to the medial aspect of the knee joint and painful points at a dose of 0.05 mJ/mm^2^ with 1000 pulses, 3 sessions per week for 2 weeks, totaling 6 sessions [20].

HILT therapy was performed using a 1064 nm wavelength Nd:YAG laser (BTL-6000 High Intensity Laser 12 W). This device has a maximum power of 12 W and 3 different treatment modes. In this study, the biostimulant and analgesic modes were used. In both modes, the application was performed with continuous circular movements. Only the analgesic mode was applied for the first 3 days. In these sessions, a total of 300 J was applied at a frequency of 25 Hz and 12 J/m^2^ to an area of 25 cm^2^. The biostimulant mode was applied starting from the fourth session. In this mode, a total of 3000 J was applied at 120 J/cm^2^. A total of 10 treatment sessions were administered over 2 weeks [13].

All participants were prescribed a standardized home exercise program aimed at improving knee joint mobility, muscle strength, and functional capacity. The program consisted of quadriceps strengthening exercises (including isometric quadriceps contractions and straight leg raises), active range-of-motion exercises for the knee joint, and stretching exercises for the hamstring and calf muscles. Exercises were performed once daily, with each session lasting approximately 20–30 min throughout the study period. Correct exercise performance was demonstrated by a physiotherapist. Adherence to the home exercise program was monitored through patient self-report during follow-up visits. Patients were asked to confirm the frequency and regularity of exercise performance at each assessment point.

### Statistical analysis

For comparing demographic characteristics between groups, the *t*-test was used for numerical variables and chi-square analysis for categorical variables. The Shapiro–Wilk test was used to determine whether the groups followed a normal distribution. The Shapiro–Wilk test is one of the most powerful tests and is provided as C in the SPSS program output; therefore, this test was chosen for application [21]. Repeated measures analysis of variance was used to compare the values obtained within the study groups according to measurement times. The *t*-test was used to compare the results obtained at different measurement times between groups. In addition, Pearson’s correlation coefficient was used to calculate the correlations of the variables belonging to the measurement times. The analyses were performed using the SPSS 22.0 program. The significance level was set at *p* < 0.05.

## Results

Fifty (83.7%) of the patients participating in the study were female, and 10 (16.3%) were male. The mean age of the patients participating in the study was 59.37 ± 8.3 years, and the mean body mass index (BMI) was 32.46 ± 4.25 kg/m^2^. When the two groups were compared, there were no significant differences in terms of gender, age, or BMI (*p* > 0.05). No statistically significant differences were found in the comparison of VAS, WOMAC, and Lequesne scores in the pre-treatment clinical and functional evaluation of the groups (*p* > 0.05) (Table 1).

**Table 1:** Comparison of clinical and sociodemographic characteristics of groups before treatment

	ESWT (*n* = 30)	HILT (*n* = 30)	Mean difference (95% CI)	*p* value	Effect size
Age (years)	59.61 ± 8.48	59.14 ± 8.12	0.475 (− 3.821 to 4.771)	0.826	0.057
Gender (female/male)	25/5	25/5		0.908	0.027
Body mass index (kg/m^2)^	32.37 ± 4.01	32.56 ± 4.5	− 0.189 (− 2.387 to 2.009)	0.864	0.044
VAS	7 ± 1	7 ± 1	0.659 (− 0.001 to 1.318)	1.00	0.516
WOMAC-pain	12.52 ± 2.57	11.34 ± 2.82	1.171 (− 0.221 to 2.563)	0.098	0.435
WOMAC-stiffness	3.90 ± 1.47	4.31 ± 1.23	− 0.407 (− 1.109 to 0.295)	0.251	0.300
WOMAC-physical function	37.35 ± 8.89	38.28 ± 11.12	− 921 (− 6.105 to 4.263)	0.723	− 0.092
WOMAC-total	56.64 ± 11.72	56.13 ± 14.68	0.506 (− 6.337 to 7.349)	0.883	0.468
Lequesne	12.10 ± 2.84	11.90 ± 2.54	0.200 (− 1.197 to 1.597)	0.775	0.074
	Group	*n*	Mean/number	*p* value	
Age (years)	ESWT	30	59.61 ± 8.48	0.826	
	HILT	30	59.14 ± 8.12		
Gender (female/male)	ESWT	30	25/5	0.908	
	HILT	30	25/5		
Symptom duration (months)	ESWT	30	40.2 ± 21.9	0.151	
	HILT	30	46.8 ± 24.0		
Body mass index (kg/m^2^)	ESWT	30	32.37 ± 4.01	0.864	
	HILT	30	32.56 ± 4.5		
VAS	ESWT	30	7 ± 1	0.058	
	HILT	30	7 ± 1		
WOMAC-pain	ESWT	30	12.52 ± 2.57	0.098	
	HILT	30	11.34 ± 2.819		
WOMAC-stiffness	ESWT	30	3.90 ± 1.47	0.251	
	HILT	30	4.31 ± 1.23		
WOMAC-physical function	ESWT	30	37.35 ± 8.89	0.723	
	HILT	30	38.28 ± 11.12		
WOMAC-total	ESWT	30	56.64 ± 11.72	0.883	
	HILT	30	56.13 ± 14.68		
Lequesne	ESWT	30	12.10 ± 2.84	0.775	
	HILT	30	11.90 ± 2.54		

*WOMAC* Western Ontario and McMaster Universities Arthritis Index, *VAS* Visual Analog Scale, *ESWT* extracorporeal shock wave therapy, *HILT* high-intensity laser therapy

When comparing pre- and post-treatment (2nd week) in the ESWT group, a significant decrease was observed in VAS, WOMAC-pain, WOMAC-stiffness, WOMAC-physical function, WOMAC-total, and Lequesne index scores (*p* < 0.05). In the ESWT group, a significant improvement was observed in all parameters when comparing post-treatment (2nd week) and the 6th week (*p* < 0.05) (Table 2).

**Table 2:** Comparison of pre-treatment and post-treatment results for the groups

	ESWT		HILT		*p*
	Mean ± SD	*p*	Mean ± SD	*p*	
**VAS**					
Pre-treatment	7 ± 1		7 ± 1		0.058
2 weeks	6 ± 1		6 ± 1		0.721
6 weeks	5 ± 2	0.001	5 ± 2	0.001	0.636
**WOMAC-pain**					
Pre-treatment	12.52 ± 2.57		11.34 ± 2.819		0.098
2 weeks	9.90 ± 1.83		9.17 ± 2.817		0.235
6 weeks	9.00 ± 2.49	0.001	8.76 ± 3.214	0.001	0.745
**WOMAC-stifness**					
Pre-treatment	3.90 ± 1.47		4.31 ± 1.23		0.251
2 weeks	3.26 ± 1.67		3.34 ± 1.17		0.818
6 weeks	2.97 ± 1.45	0.001	3.21 ± 1.18	0.001	0.487
**WOMAC-physical function**					
Pre-treatment	37.35 ± 8.89		38.28 ± 11.12		0.723
2 weeks	31.65 ± 8.04		32.76 ± 9.93		0.634
6 weeks	28.39 ± 6.80	0.001	29.00 ± 10.76	0.001	0.792
**WOMAC-total**					
Pre-treatment	56.64 ± 11.72		56.13 ± 14.68		0.883
2 weeks	46.57 ± 10.03		46.77 ± 13.16		0.948
6 weeks	41.98 ± 8.84	0.001	43.10 ± 14.03	0.001	0.711
**Lequesne**					
Pre-treatment	12.10 ± 2.84		11.90 ± 2.54		0.775
2 weeks	9.97 ± 2.01		10.03 ± 2.63		0.912
6 weeks	9.13 ± 1.96	0.001	9.14 ± 2.71	0.001	0.988

*WOMAC* Western Ontario and McMaster Universities Arthritis Index, *VAS* Visual Analog Scale, *ESWT* extracorporeal shock wave therapy, *HILT* high-intensity laser therapy

In the HILT group, a significant decrease was observed in VAS, WOMAC pain, WOMAC stiffness, WOMAC physical function, WOMAC total, and Lequesne index scores when comparing pre-treatment and post-treatment (2nd week) (*p* < 0.05). In the HILT group, significant improvement was observed in all parameters when comparing post-treatment (2nd week) and 6th week (*p* < 0.05) (Table 2).

When comparing the HILT and ESWT groups, no significant differences were found in VAS, WOMAC pain, WOMAC stiffness, WOMAC physical function, WOMAC total, and Lequesne index scores before treatment and after treatment (2nd week) (*p* > 0.05). Similarly, no significant differences were found in any parameters when comparing post-treatment (2nd week) and 6th week (*p* > 0.05) (Table 2).

## Discussion

In this study, we aimed to compare the effectiveness of ESWT and HILT treatments on parameters such as pain and function in patients with knee OA. The results of our study revealed that both treatment methods provided significant improvements in key objectives such as pain control, joint stiffness, and increased functional capacity in knee OA.

The goal of treatment in knee OA is to reduce morning stiffness and pain, preserve joint range of motion and muscle strength by stopping further joint damage, educate the patient about OA, and improve quality of life by minimizing dependence in daily living activities [22]. Non-pharmacological treatment methods include physical therapy, education, weight loss, thermal modalities, and transcutaneous electrical nerve stimulation. ESWT is used in the treatment of musculoskeletal disorders and is also widely used in conditions such as plantar fasciitis, lateral epicondylitis, and fracture healing [23]. In recent years, researchers have investigated the effects of ESWT on knee osteoarthritis, and research results have shown that it may be effective in knee osteoarthritis [24]. ESWT induces vasodilation by stimulating nitric oxide release, causes angiogenesis by stimulating microcirculation in blood and lymph vessels, and produces an anti-inflammatory effect along with increased metabolism and growth factor release. It increases cell permeability. It has been suggested that ESWT reduces pain by decreasing the release of local pain factors such as substance P and by damaging unmyelinated sensory fibers [25]. In a rat OA knee model, ESWT improved the rat’s walking ability and reduced Calcitonin Gene Related Peptide (CGRP) positive neurons in the dorsal root ganglia [26]. Extracorporeal shock wave therapy improves motor dysfunction and pain originating from knee osteoarthritis in rats [26]. ESWT affects inflammatory processes and initiates bone repair. It also activates numerous cellular processes necessary for tissue regeneration and neovascularization [27]. Our study also demonstrated that ESWT significantly reduced pain, WOMAC-pain, WOMAC-stiffness, WOMAC-physical function, and WOMAC-total scores in patients with knee osteoarthritis. ESWT histologically stimulates subchondral bone anabolism and promotes trabecular micro-healing. According to immunohistochemical analysis, ESWT causes a decrease in carboxy-terminal telopeptide type II collagen (CTX-II) concentration and matrix metalloproteinase (MMP) expression in rats with knee OA, indicating a decrease in cartilage catabolism [28]. In our study, ESWT also provided significant improvements in pain, stiffness, and function parameters both in the short term and at 6 weeks. These findings are consistent with the analgesic and tissue repair-promoting effects of ESWT.

HILT is a treatment method that has been increasingly applied in physical therapy practice. This device is a non-invasive treatment method that does not cause pain in the patient. With its high penetration power, it is effective in deep areas and large joints that low-intensity laser therapy cannot reach. Studies have demonstrated that high-intensity laser therapy activates oxidative processes, increases energy production, and exhibits biostimulant properties. The efficacy of HILT has been proven in the treatment of many musculoskeletal disorders, and HILT has anti-inflammatory, anti-edema, analgesic, and reparative effects [29]. In a study by Kheshie et al. [30] evaluating the effectiveness of laser therapy in 53 OA patients, both low and high-intensity laser therapy provided significant improvement in VAS and WOMAC scores, with high-intensity laser therapy being more effective. Similarly, Ciplak et al. [31] evaluated the efficacy of HILT and conventional treatment (hot pack, TENS, and ultrasound) in 48 cases of knee osteoarthritis and showed improvement in pain and functional scales in both groups. The authors stated that HILT was more effective than conventional therapy and that this effect persisted at 6 weeks [31]. Similarly, in our study, we observed significant improvement in pain, stiffness, and functional status at the 6-week follow-up. The analgesic effect of HILT is based on properties such as slowing the transmission of pain stimuli and increasing the production of opioid substances in the body. HILT also stimulates healing by increasing blood flow, vascular permeability, and cell metabolism [32]. In addition to its analgesic effect, HILT may contribute to pain and functional improvement by supporting cartilage repair through its biostimulant effect on chondrocytes [33, 34].

There are a limited number of studies in the literature comparing the efficacy of ESWT and HILT. Mostafa et al. [35] compared the efficacy of HILT and ESWT in patients with knee OA and concluded that HILT was more effective than ESWT. HILT was administered 3 days a week for 4 weeks, and ESWT was administered once a week for 4 weeks. In our study, ESWT was administered 3 times a week for a total of 6 sessions, which may explain the difference in our results. Li et al. [36] retrospectively evaluated 105 knee OA patients. Sixty patients received ESWT, while 45 patients received laser therapy, and they reported that ESWT was superior to laser therapy at 6 and 12 weeks. However, the retrospective design of this study, along with the lack of clarity regarding details such as treatment doses and application duration, limits the generalizability of the results. There is still no consensus in the literature regarding the application methods, devices, and doses of ESWT and HILT. ESWT is dose-dependent, and the optimal energy level and treatment duration have not been determined [20].

This study shows that ESWT and HILT have similar effects on pain and functional improvement in knee OA. However, investigating the long-term effects of the treatments, their efficacy in different stages of OA, and placebo-controlled studies could further clarify the clinical use of these treatments. Both treatments can be considered non-invasive and safe options; however, it is important to offer individualized treatment options based on patient characteristics and clinical status. Since ESWT and HILT methods require different devices and techniques, the suitability of treatment methods for the patient may be reflected in clinical practice. The observation that both treatment methods show similar efficacy may provide flexibility in practice and allow patients to learn more about their treatment options.

Our study has some limitations. The relatively small sample size, the lack of long-term follow-ups, and the absence of a control group that did not receive treatment can be considered limitations.

Ultimately, both ESWT and HILT applications were found to be effective and safe modalities in terms of pain and functionality in knee osteoarthritis. However, no superiority of ESWT or HILT over each other was observed in the intergroup comparison. Clinicians can consider HILT and ESWT treatments as options when planning knee osteoarthritis treatment.

## Data Availability

The data used in this study will be shared upon request by the relevant researchers.
